# Polymorphism of glucocorticoid receptor gene (*rs41423247*) in functional seizures (psychogenic nonepileptic seizures/attacks)

**DOI:** 10.1002/epi4.12816

**Published:** 2023-08-24

**Authors:** Negar Firouzabadi, Ali A. Asadi‐Pooya, Nahid Alimoradi, Leila Simani, Marjan Asadollahi

**Affiliations:** ^1^ Department of Pharmacology and Toxicology, School of Pharmacy Shiraz University of Medical Sciences Shiraz Iran; ^2^ Epilepsy Research Center Shiraz University of Medical Sciences Shiraz Iran; ^3^ Jefferson Comprehensive Epilepsy Center, Department of Neurology Thomas Jefferson University Philadelphia Pennsylvania USA; ^4^ Brain Mapping Research Center Shahid Beheshti University of Medical Sciences Tehran Iran; ^5^ Department of Pharmaceutical Sciences, College of Pharmacy University of Kentucky Lexington Kentucky USA; ^6^ Department of Epilepsy, Loghman Hakim Hospital Shahid Beheshti University of Medical Sciences Tehran Iran

**Keywords:** genetic, nonepileptic, psychogenic, seizure, single nucleotide polymorphism

## Abstract

**Objective:**

We investigated the association between the glucocorticoid receptor (GR) gene, also known as the nuclear receptor subfamily 3, group C, member 1 (NR3C1), *rs41423247* polymorphism, and functional seizures (psychogenic nonepileptic seizures/attacks) in a case–control study. We hypothesized that the tested polymorphism has significant associations with functional seizures (psychogenic nonepileptic seizures/attacks) independent from comorbid depression.

**Methods:**

Seventy patients with functional seizures (psychogenic nonepileptic seizures/attacks), 70 with major depressive disorder (MDD), and 70 healthy controls (HCs) were studied. Their DNAs were analyzed for NR3C1 *rs41423247* polymorphism.

**Results:**

Genotype and allele frequencies of *rs41423247* were different between the three groups. G allele carriers were more frequent in patients with functional seizures (psychogenic nonepileptic seizures/attacks) and those with MDD compared to HCs (*p* = 0.0001). However no significant difference was observed with respect to allele distributions between functional seizures (psychogenic nonepileptic seizures/attacks) and MDD groups (*p* = 0.391). CC genotype was less often associated with functional seizures (psychogenic nonepileptic seizures/attacks) versus HC: Codominant model; *p* = 0.001, OR = 0.11, 95% CI = 0.05–0.24, and −2loglilkelihood = 231.7. In comparison between functional seizures (psychogenic nonepileptic seizures/attacks) group and other (MDD + HC) groups, we observed a significant association between CG genotype and functional seizures (psychogenic nonepileptic seizures/attacks) (Codominant model; *p* = 0.001, OR = 5.63, 95% CI = 2.60–12.40 and −2loglikelihood = 245.99).

**Significance:**

Patients with functional seizures (psychogenic nonepileptic seizures/attacks) and those with MDD were significantly more often G allele carriers in *rs41423247* compared with HCs. We observed a significant association between CG genotype and functional seizures (psychogenic nonepileptic seizures/attacks). However, we could not exclude the possibility of confounding effects of depression. Future genetic studies of patients with functional seizures (psychogenic nonepileptic seizures/attacks) should include a comparison group with depression in addition to a comparison group of HCs.

**Key points**

- Seventy patients with functional seizures (psychogenic nonepileptic seizures/attacks), 70 with MDD, and 70 HCs were studied.
- Their DNAs were analyzed for NR3C1 *rs41423247* polymorphism.
- We observed a significant association between CG genotype and functional seizures (psychogenic nonepileptic seizures/attacks).
- G allele carriers were more frequent in functional seizures (psychogenic nonepileptic seizures/attacks) group and those with MDD compared with HCs (*p* = 0.0001).
- However, no significant difference was observed with respect to allele distributions between functional seizures (psychogenic nonepileptic seizures/attacks) and MDD groups (*p* = 0.391).

## 1 INTRODUCTION

Patients with functional seizures (psychogenic nonepileptic seizures/attacks) are commonly seen at epilepsy centers and neurology clinics.^1^ Knowledge on the biological underpinnings of functional seizures (psychogenic nonepileptic seizures/attacks) is still scarce, but there is growing evidence about variations in functional and structural brain connectivity in these patients.^2^ Recently, three studies have implicated the role of genetic issues in functional seizures (psychogenic nonepileptic seizures/attacks).^3^, ^4^, ^5^ One study at our center investigated the associations between *FKBP5* (a co‐chaperone that modulates glucocorticoid receptor (GR) activity in the human brain) single nucleotide polymorphisms (SNPs) and functional seizures (psychogenic nonepileptic seizures/attacks).^5^ We observed that patients with functional seizures (psychogenic nonepileptic seizures/attacks) and also patients with depression had significantly more AA genotypes in both *rs9470080* and *rs1360780* SNPs compared with healthy controls (HCs). However, there were no significant differences between depression and functional seizures (psychogenic nonepileptic seizures/attacks) groups in terms of genotype and allelic frequencies for both SNPs.^5^ In one study, the authors observed that six (5.9%) patients with functional seizures (psychogenic nonepileptic seizures/attacks) carried pathogenic / likely pathogenic variants.^3^ In another study, the authors suggested that in patients with functional seizures (psychogenic nonepileptic seizures/attacks), there was significant correlation with genes that are over‐represented in adrenergic, serotonergic, oxytocin, opioid, and GABA receptor signaling pathways.^4^

One previous study showed that patients with functional seizures (psychogenic nonepileptic seizures/attacks) had significantly increased basal diurnal cortisol levels compared to HCs. The increased basal diurnal cortisol levels in these patients were not explained by depression.^6^ A recent study suggested that hypothalamic–pituitary–adrenal (HPA) axis dysregulation and changes in glucocorticoid (cortisol) signaling at the molecular level may contribute to increased vulnerability for functional neurological disorder (FND) symptoms.^7^ However, patients with functional movement disorders (*n* = 33) did not differ from age‐ and sex‐matched controls (*n* = 33) with respect to levels of circulating cortisol in one study.^8^ On the other hand, a dysfunction of the HPA axis is closely related to the occurrence of some psychiatric disorders (e.g., depression).^9^ The GR, also known as the nuclear receptor subfamily 3, group C, member 1 (NR3C1), provides negative feedback to the HPA axis. A meta‐analysis showed that the NR3C1 *rs41423247* homozygous mutation may be a risk factor for depression.^9^ Another study showed that polymorphisms in genes that are important for the HPA axis (e.g., *rs41423247* in the NR3C1 gene) are associated with self‐assessed anxiety in healthy individuals in a gender‐specific (female) manner.^10^

Considering the observation that patients with functional seizures (psychogenic nonepileptic seizures/attacks) have significantly increased basal diurnal cortisol levels,^6^ the potential role of HPA axis in FND,^7^ the observation that NR3C1 gene provides negative feedback to the HPA axis,^9^ and the observed associations between *rs41423247* in the NR3C1 gene and psychopathologies,^9^, ^10^ we aimed to investigate the association between NR3C1 *rs41423247* polymorphism and functional seizures (psychogenic nonepileptic seizures/attacks) in a case–control study. We hypothesized that the tested polymorphism has significant associations with functional seizures (psychogenic nonepileptic seizures/attacks) independent from comorbid depression in these patients. While a candidate gene approach limits the likelihood of a discovery, a well‐selected candidate gene (see the above explanation) can look for pathological processes in modestly sized cohorts by focusing on a limited number of pathways with higher neurobiological relevance with the target condition.^11^ Adopting the above strategy, other candidate genes that are implicated in the HPA axis and psychopathologies could also be tested in the future.

## 2 METHODS

### 2.1 Participants and study design

This was a cross‐sectional case–control study. Seventy patients with functional seizures (psychogenic nonepileptic seizures/attacks), who were admitted at Loghman Hakim Hospital, Tehran, Iran or at Shiraz Comprehensive Epilepsy Center at Shiraz University of Medical Sciences, Shiraz, Iran, from 2020 until 2021, were studied. These were the same patients as in our previous study.^5^ Also, 70 individuals diagnosed with major depressive disorder (MDD) (diagnosed and confirmed by a psychiatrist according to the DSM‐V criteria who referred to Hafiz Hospital, Shiraz, Iran, from January 2020 until September 2020), and 70 HCs (healthy people with no history of seizures and no history of psychiatric disorders) were included. Patients with a documented diagnosis of functional seizures (psychogenic nonepileptic seizures/attacks), determined by clinical assessment and video‐EEG monitoring with ictal recording, were included. We always make a documented diagnosis of functional seizures (psychogenic nonepileptic seizures/attacks) if history is compatible with the diagnosis; habitual events are witnessed by the epileptologist, showing semiology typical of functional seizures (psychogenic nonepileptic seizures/attacks) while on video‐EEG monitoring; and finally, no epileptiform activity is detected immediately before, during, or after the attack that has been captured during video‐EEG recording.^12^ The epileptologists interviewed all these patients. Patients with comorbid epilepsy were excluded. The Persian‐validated Hospital Anxiety and Depression Scale (HADS) was used in the functional seizures (psychogenic nonepileptic seizures/attacks) group (with a cutoff point of 8 for depression).^13^

Due to significant associations between depression and functional seizures (psychogenic nonepileptic seizures/attacks), two control groups (i.e., MDD and HC groups) were included in the study in order to assess the potential confounding effects of depression. Enrolled individuals were between 18 and50 years of age. The written consent was obtained from the participants prior to the enrollment. All patients were of Caucasian origin. The study was approved by the Research Ethics Committee of Shiraz University of Medical Sciences, Shiraz, Iran (98‐01‐106‐21304).

### 2.2 DNA extraction and genotyping

Blood sample was collected from each participant in an EDTA tube, and genomic DNA was extracted from whole blood using a slating out method.^14^ The quality of the extracted DNA was assessed using NanoDrop®. Polymerase chain reaction (PCR) amplification of *rs41423247* was carried out using primers mentioned in the Table S1. Analysis of the SNP polymorphism of *rs41423247* was carried out via the PCR‐restricted fragment length polymorphism (PCR‐RFLP) method. Genotyping of *rs41423247* was carried out as previously described.^15^, ^16^, ^17^ A total of 50 ng genomic DNA was mixed with 0.5 pmol of each PCR primer in a total volume of 25 μl containing 12.5 μl Master Mix (2X) (Ampliqon, Denmark). PCR cycling conditions were as follows: an initial denaturation at 94°C for 5 min, followed by 30 cycles at 94°C for 30 s, 55°C for 45 s, and 72°C for the 45 s, and a final extension at 72°C for 7 min. After PCR amplification, 10 μl of PCR products of rs41423247 polymorphism was incubated with 2 μl of BclI restriction endonuclease (Fermentas, Lithuania) for 16 h at 37°C. Digested fragments were separated by electrophoresis on a 3% agarose gel (Invitrogen® Ultra‐Pure) and visualized in a UV transilluminator.

### 2.3 Statistical analysis

Data were analyzed using SPSS Statistics software (23.0; SPSS Inc.). Quantitative variables are demonstrated as mean ± standard deviation (SD) and categorical data are shown in percentage (%). Chi‐square (χ^2^) test was used to compare categorical values. Continuous variables were compared between groups using one‐way ANOVA test. Logistic regression analysis was performed to assess different inheritance models. Adjusted *p*‐values, odds ratio (OR) and 95%confidence interval (CI) were calculated using multiple logistic regression analysis. A *p* value <0.05 was considered as statistically significant.

## 3 RESULTS

No significant differences were found in the main demographic variables between the groups [i.e., female sex functional seizures (psychogenic nonepileptic seizures/attacks): 43, MDD: 51, HC: 47], *p* = 0.355; mean age (SD) [functional seizures (psychogenic nonepileptic seizures/attacks): 32.6 (9.5), MDD: 34.0 (9.6), HC: 34.3 (6.5), *p* = 0.243]. Forty‐eight patients (68.6%) from the functional seizures (psychogenic nonepileptic seizures/attacks) group had depression based on their HADS scores.

Genotype and allele frequencies of *rs41423247* in the studied groups are presented in Table 1, with the major allele frequency of 52.9% in the functional seizures (psychogenic nonepileptic seizures/attacks) group. Genotype distributions in functional seizures (psychogenic nonepileptic seizures/attacks) and HC groups were in Hardy‐Weinberg‐Equilibrium (HWE). The *p*‐values for HWE in functional seizures (psychogenic nonepileptic seizures/attacks) and HC groups were 0.483 and 0.3887, respectively. However, the *p*‐value for MDD group was <0.05, which shows lack of agreement with HWE.

**TABLE 1:** Genotype and allele frequencies of *rs41423247* in functional seizures (psychogenic nonepileptic seizures/attacks), MDD, and HC groups (% in parenthesis).

	FS (*n* = 70)	MDD (*n* = 70)	HC (*n* = 70)	*p*c	Chi‐square
*rs41423247*					
Genotype					
CC	11 (15.7)	20 (28.6)	42 (60.0)	0.0001	66.49
CG	37 (52.9)	11 (15.7)	26 (37.1)		
GG	22 (31.4)	39 (55.7)	2 (2.9)		
Allele					
C	59 (42.2)	51 (36.4)	110 (78.6)	0.0001	58.67
G	81 (57.8)	89 (63.6)	30 (21.4)		

Abbreviations: FS, Functional seizures (psychogenic nonepileptic seizures/attacks); HC, Healthy control; MDD, Major depressive disorder; *p*c, *p*‐value for Chi‐square test.

As shown in Table 1, the genotype distributions were different between the groups. Additionally, G allele carriers were more frequent in patients with functional seizures (psychogenic nonepileptic seizures/attacks) and those with MDD compared to HCs. As demonstrated in Table 1, CG genotype was more frequent in patients with functional seizures (psychogenic nonepileptic seizures/attacks) compared to other genotypes and other study groups (MDD and HC). In the HC group, CC genotype was more frequent both compared with the other genotypes and also compared with the genotype distributions in the other study groups [functional seizures (psychogenic nonepileptic seizures/attacks) and MDD]. Allele distributions confirmed a significantly higher frequency of the risk allele (G allele) in functional seizures (psychogenic nonepileptic seizures/attacks) and MDD groups compared with the HC group.

The majority of MDD patients were homozygote for the risk allele (GG), whereas in functional seizures (psychogenic nonepileptic seizures/attacks) group the majority of patients were heterozygote for the risk allele (CG). Lastly, HC were mostly homozygote for the major allele (CC) (Table 1).

We applied the logistic regression analysis in order to study various inheritance models. This test showed that CC genotype was less often associated with functional seizures (psychogenic nonepileptic seizures/attacks) (functional seizures [psychogenic nonepileptic seizures/attacks] vs. HC: Codominant model; *p* = 0.001, OR = 0.11, 95% CI = 0.05–0.24, and −2loglilkelihood = 231.7). In comparison between functional seizures (psychogenic nonepileptic seizures/attacks) group and other (MDD + HC) groups, we observed a significant association between CG genotype and functional seizures (psychogenic nonepileptic seizures/attacks) (Codominant model; *p* = 0.001, OR = 5.63, 95% CI = 2.60–12.40 and −2loglikelihood = 245.99). Comparing functional seizures (psychogenic nonepileptic seizures/attacks) and MDD groups, we observed that patients with GG genotype less often had functional seizures (psychogenic nonepileptic seizures/attacks) (Recessive model; *p* = 0.001, OR = 0.038, 95% CI = 0.01–0.16 and −2loglilkelihood = 220.7). In comparison between functional seizures (psychogenic nonepileptic seizures/attacks) group and MDD group, genotype distributions were different (*p* = 0.006); however, no significant difference was observed with respect to allele distributions (*p* = 0.391).

The number of the patients with functional seizures (psychogenic nonepileptic seizures/attacks), who did not have depression (*N* = 22), was too small for any reliable statistical analyses (in patients with functional seizures [psychogenic nonepileptic seizures/attacks] with or without depression); however, as shown in Table 2, there were no significant differences in genotype and allele distributions of *rs41423247* in patients with functional seizures (psychogenic nonepileptic seizures/attacks), with and without depression. Among patients with functional seizures (psychogenic nonepileptic seizures/attacks), 49 individuals (70%) had generalized motor events and the remaining 21 (30%) had akinetic events; genotype distributions were not different between the two semiologies (*p* = 0.274).

**TABLE 2:** Genotype and allele frequencies of *rs41423247* in patients with functional seizures (psychogenic nonepileptic seizures/attacks) with and without depression (% in parenthesis) and the associated *p*‐values.

	FS^+^ (*n* = 48)	FS^−^ (*n* = 22)	*p*c	Chi‐square
*rs41423247*				
Genotype				
CC	7 (14.6)	4 (18.2)	0.092	4.78
CG	22 (45.8)	15 (68.2)		
GG	19 (39.6)	3 (13.6)		
Allele				
C	36 (37.5)	23 (52.3)	0.100	2.7
G	60 (62.5)	21 (47.7)		

Abbreviations: FS, Functional seizures (psychogenic nonepileptic seizures/attacks); FS^+^, Functional seizures with depression; FS^−^, Functional seizures without depression; *p*c, *p*‐value for Chi‐square test.

## 4 DISCUSSION

In the current study, we investigated the GR gene polymorphism (*rs41423247*) in Iranian patients with functional seizures (psychogenic nonepileptic seizures/attacks). We observed that patients with functional seizures (psychogenic nonepileptic seizures/attacks) and those with MDD were significantly more often G allele carriers in *rs41423247* compared with those in HCs. We also observed a significant association between CG genotype and functional seizures (psychogenic nonepileptic seizures/attacks). While HPA axis dysregulation and changes in glucocorticoid signaling at the molecular level may be involved in FND symptoms,^7^, ^18^ the biological explanations of this association between the GR gene polymorphism (*rs41423247*) and functional seizures (psychogenic nonepileptic seizures/attacks) should be explored in future studies. As MDD + HC were taken together as the control group, one could argue that it controls to some extent for depression and CG genotype might be specific to functional seizures (psychogenic nonepileptic seizures/attacks). However, in comparison between functional seizures (psychogenic nonepileptic seizures/attacks) group and MDD group, while genotype distributions were different, no significant difference was observed with respect to allele distributions. In other words, we could not completely exclude the possibility of confounding effects of depression with regards to the association between *rs41423247* polymorphism and functional seizures (psychogenic nonepileptic seizures/attacks), probably due to our small sample size. In spite of this, the hypothesis of this association (between GR gene SNPsand functional seizures [psychogenic nonepileptic seizures/attacks]) deserves more attention in larger studies in the future. Furthermore, this observation highlights the need to include a comparison group with depression in any future genetic study of patients with functional seizures (psychogenic nonepileptic seizures/attacks).^5^

The evidence on the genetic underpinnings of functional seizures (psychogenic nonepileptic seizures/attacks) is so far scarce in the literature. In spite of this, the hypothesis of a genetic etiology for functional seizures (psychogenic nonepileptic seizures/attacks) is plausible based on the emerging evidence on altered functional and structural brain connectivity in patients with functional seizures (psychogenic nonepileptic seizures/attacks) compared with those in healthy individuals.^2^ The evidence shows that brain connectivity is under genetic influence.^19^ In addition, there is strong evidence in the literature on the genetic basis of other stress‐associated neuropsychological disorders [e.g., post‐traumatic stress disorder (PTSD)].^20^ Functional seizures (psychogenic nonepileptic seizures/attacks) and other stress‐associated neuropsychological disorders share a common core of clinical characteristics; therefore, it is reasonable to investigate whether variants in stress‐related genes (e.g., GR gene, FKBP5) also contribute to the development of various FNDs, including functional seizures (psychogenic nonepileptic seizures/attacks). However, it is unlikely that any of these gene variations be specific to functional seizures (psychogenic nonepileptic seizures/attacks) or other FNDs; they may represent genetic susceptibilities that combined with life stressors and other risk factors may lead to individual FND phenotypes.^5^

### 4.1 Limitations

Our study has some significant limitations. First, it included a small sample size. Second, we did not clinically assess a comorbid diagnosis of MDD in those with functional seizures (psychogenic nonepileptic seizures/attacks) (we used HADS). In addition, we did not consider other potentially important confounding variables such as PTSD. Furthermore, this study was based on a single country which may limit its generalizability to other populations. The findings in this study have not been tested in a separate replication cohort; thus, the results should be viewed as exploratory. Finally, our analyses tested *rs41423247* in the NR3C1 gene; other alterations were not investigated and we cannot ascertain a complete coverage of this important gene.

## 5 CONCLUSION

Patients with functional seizures (psychogenic nonepileptic seizures/attacks) and those with MDD were significantly more often G allele carriers in *rs41423247* compared with HCs; CG genotype was more frequent in patients with functional seizures (psychogenic nonepileptic seizures/attacks). However, we could not completely exclude the possibility of confounding effects of depression. Future genetic studies of patients with functional seizures (psychogenic nonepileptic seizures/attacks) should include a comparison group with depression in addition to a comparison group of HCs.

## AUTHOR CONTRIBUTIONS

Ali A. Asadi‐Pooya: Study conceptualization and design, data collection, and manuscript preparation. Negar Firouzabadi: Data collection, statistical analyses, and manuscript preparation. Others: Data collection and manuscript preparation.

## FUNDING INFORMATION

This research was partially supported by grants from the Shiraz University of Medical Sciences. The funding sources had no involvement in study design; in the collection, analysis, and interpretation of data; in the writing of the report; and in the decision to submit the article for publication.

## CONFLICT OF INTEREST STATEMENT

Ali A. Asadi‐Pooya: Honoraria from Cobel Daruo; Royalty: Oxford University Press (Book publication); Grant from the National Institute for Medical Research Development. Marjan Asadollahi: honorarium from Cobel Darou, Actoverco, and Sanofi; Others: no conflict of interest.

## ETHICS STATEMENT

The study was approved by the Research Ethics Committee of Neuroscience Research Center, Shahid Beheshti University of Medical Sciences, Tehran, Iran (IR.SBMU.PHNS.REC.1399.007). All the participants gave their written informed consent before participating in the study. Consent for publication is not applicable to this study.

## Supporting information


Table S1.

## Data Availability

The data used in this study will be shared as per regulations of the Shiraz University of Medical Sciences. To obtain these data, one should contact the vice chancellor for research, Shiraz University of Medical Sciences (project number: 98‐01‐106‐21304).
