# Supplementary material for: Polymorphism of glucocorticoid receptor gene (rs41423247) in functional seizures (psychogenic nonepileptic seizures/attacks)
Source: Epilepsia Open. 2023 Aug 24;8(4):1425–31. doi: 10.1002/epi4.12816 (PMC10690659; doi:10.1002/epi4.12816)
Supplement: Supplementary file 1 — Table S1. [file EPI4-8-1425-s001.docx]

**Supplementary Table 1.** List of forward and reverse primers for PCR-RFLP of *rs41423247* and its associated restriction enzyme and DNA fragment sizes.

| Polymorphism | Primer sequence (5′-3′) | TA ^(°C)^ | Restriction enzyme | DNA fragment size (bp) |
| --- | --- | --- | --- | --- |
| *NR3C1* (*rs41423247*) | F- TGCTGCCTTATTTGTAAATTCGT  R- AAGCTTAACAATTTTGGCCAT | 55 | *NR3C1*  16 hrs/37°C | 335/222/117 |
| TA: Annealing temperature, bp: base pair | | | | |
